# Exploring the Rare Etiology of Severe Anemia in an Immunocompromised Patient

**DOI:** 10.7759/cureus.16750

**Published:** 2021-07-30

**Authors:** Keerthy D Joseph, Vihitha Thota, Ashish Bains, Neel S Patel, Ruqqiya Mustaqeem, Sana Mulla, Rajesh Thirumaran, Jakub Trawinski

**Affiliations:** 1 Internal Medicine, Mercy Catholic Medical Center, Darby, USA; 2 Hematopathology, Temple University Hospital, Philadelphia, USA; 3 Hematology/Oncology, Mercy Catholic Medical Center, Darby, USA; 4 Internal Medicine, Thomas Jefferson University, Philadelphia, USA

**Keywords:** pure red cell aplasia, b19 parvovirus infection, intravenous immunoglobulin (ivig), impaired erythropoiesis, bone marrow biopsy, immunohistochemical stain

## Abstract

Pure red cell aplasia (PRCA) is a rare cause of profound anemia, marked by very low reticulocyte count and near to complete absence of erythroid precursor cells in the bone marrow. PRCA can be congenital such as in the case of children with Diamond- Blackfan anemia or acquired, which is often triggered by exposure to certain viruses or drugs. Management depends on the underlying etiology of PRCA. Here, we present the case of a young male with underlying acquired immunodeficiency syndrome, who presented with a hemoglobin of 2.6 g/dL, initially thought to be secondary to gastrointestinal blood loss, but was later discovered to have parvovirus-induced PRCA.

## Introduction

In pure red cell aplasia (PRCA), red cell production is either almost or completely impaired; the antigen expressed on an early erythroid precursor or progenitor cell is often the target of attack. Primary PRCA is an idiopathic autoimmune disorder in which either autoantibodies or T lymphocytes suppress erythropoiesis [1,2]. Secondary PRCAs can occur due to exposure to drugs (such as phenytoin or trimethoprim-sulfamethoxazole), viral infections (i.e., B19 parvovirus, human immunodeficiency virus [HIV], or Epstein-Barr virus), immune disorders such as systemic lupus erythematosus or rheumatoid arthritis, or neoplasms (e.g. multiple myeloma, Hodgkin's lymphoma) [1,2]. 

Parvovirus causes PRCA by directly attacking and destroying proerythroblasts after attaching to the group P antigen receptor [3,4]. Transient anemia secondary to parvovirus infection occurs in immunocompetent individuals with underlying hemolytic anemia, whereas chronic B19 parvovirus infection occurs in severely immunocompromised individuals (i.e., organ transplant or HIV patients) [4].

## Case presentation

A 38-year-old African American male with a past medical history of acquired immunodeficiency syndrome, noncompliant with antiretroviral therapy (ART) regimen and last CD4 count unknown, was recommended to come to the hospital after outpatient labs revealed a low hemoglobin (Hgb). On admission, patient reported one week of melanotic stools, generalized weakness, and progressively worsening shortness of breath. Patient was tachycardic to 106 beats per minute (bpm) on arrival; all other vitals were stable. Labs revealed a Hgb of 2.6 g/dL, hematocrit of 7.5%, mean corpuscular volume of 95.4 fL, and low reticulocyte percentage of 0.2. Liver function tests, leukocyte count, platelet count, vitamin B12, and folate values were within normal limits. Iron panel was suggestive of anemia of chronic disease with elevated ferritin level (709 ng/mL) and low total iron-binding capacity level (174 ug/dL). Coagulation studies and hemolysis labs (i.e., lactate dehydrogenase and haptoglobin) were unremarkable. Computed tomography angiography of abdomen and pelvis was negative for any actively bleeding lesion. Patient was started on a proton pump inhibitor (PPI) drip and admitted for presumed acute blood loss anemia secondary to gastrointestinal (GI) bleed, for which four units of blood were transfused.

**Figure 1:**
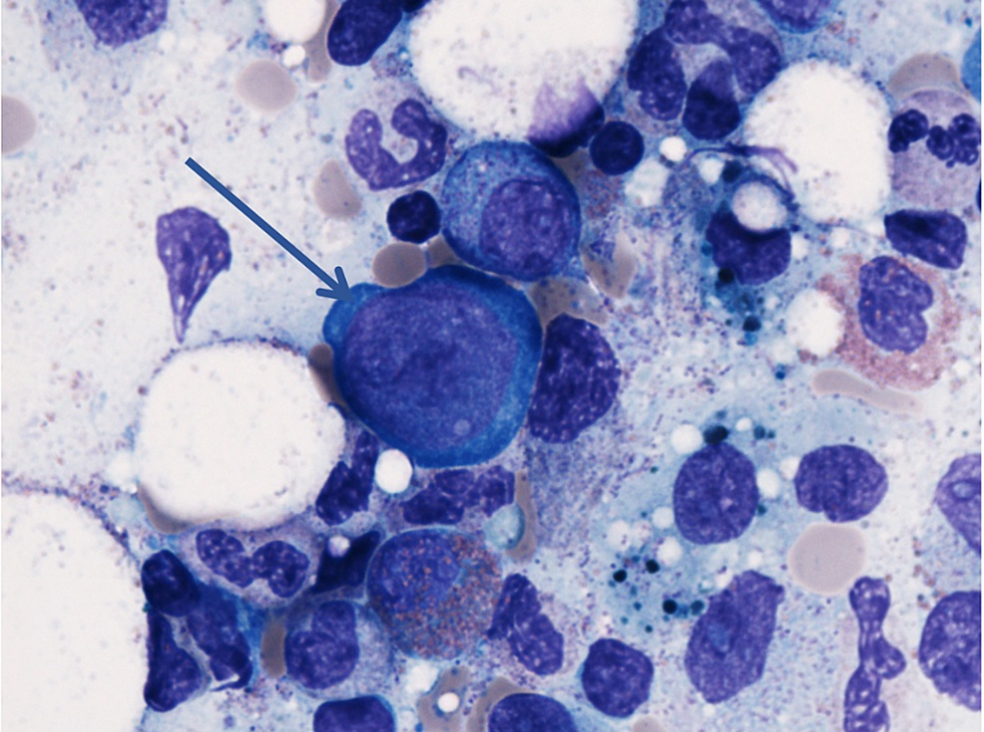
Giant pronormoblasts, characteristic of parvovirus infection (100x magnification)

**Figure 2:**
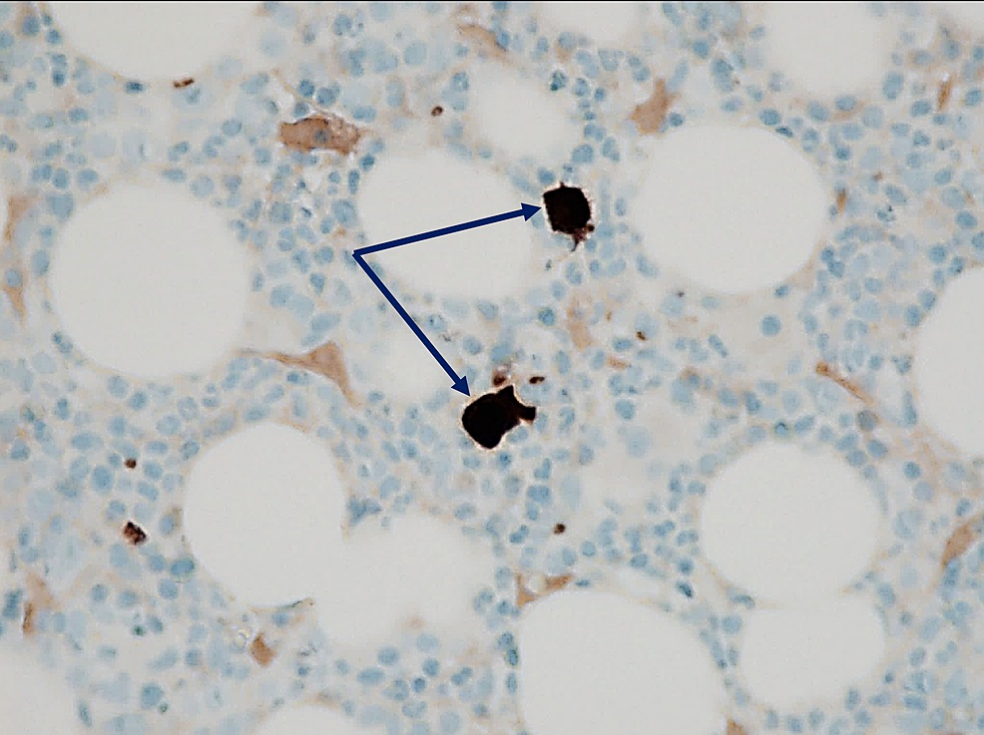
Immunohistochemistry staining positive for parvovirus B19 antigens in scattered giant pronormoblasts (40X magnification)

Esophagogastroduodenoscopy during hospital stay revealed gastritis; gastric biopsy revealed chronic active *Helicobacter pylori-*associated gastritis, for which patient was started on a 14-day course of clarithromycin, amoxicillin, and PPI. Few days into hospitalization, CD4 count resulted as 37. Given the patient’s history of immunocompromised state and severely low Hgb with reticulocytopenia on presentation, a bone marrow biopsy was performed to evaluate for potential hypoproliferative etiology. It revealed a hypercellular bone marrow with near-absent erythroid precursors and scattered giant pronormoblasts (Figure 1), consistent with PRCA. Although quantitative polymerase chain reaction (PCR) testing for parvovirus B19 DNA was negative, immunohistochemical (IHC) staining was positive for parvovirus B19 capsid proteins (Figure 2), with overall pathology consistent with parvovirus-induced PRCA. Patient was started on intravenous immunoglobulin (IVIG) for a total five-day course as per hematology recommendations with stabilization of Hgb at 8.0 g/dL on the day of discharge. Patient was discharged home with instructions to comply with ART, and during a follow-up two months later, patient was doing well clinically with stable Hgb levels.

## Discussion

PRCA is an extremely rare cause of severe anemia with an estimated prevalence of 5 per million individuals [5]. PRCA diagnosis is established when all of the following criteria are met: 1. Normocytic, normochromic anemia. 2. Absolute reticulocyte count is less than 10,000/microliter 3. Normal white blood cell and platelet counts. 4. Normocellular bone marrow with erythroblasts totaling less than 1%. 5. No significant abnormalities in the myeloid, lymphocytic, or megakaryocyte lineages [1,2]. Clinically, PRCA has an insidious presentation; most individuals are asymptomatic until the Hgb is severely reduced with manifestations of significant pallor and decreased exercise tolerance. In terms of labs, anemia is often severe with Hgb less than 4 g/dL and reticulocytes are often absent or markedly decreased. Other cell lines (leukocytes and platelets) are unchanged, unless patient has an underlying hematologic disorder [1]. Individuals who are taking a drug commonly associated with PRCA should stop taking the drug and be monitored closely for about four to six weeks.

Bone marrow biopsy, followed by routine IHC staining, is crucial in all cases of suspected PRCA. Bone marrow biopsy will often indicate whether the patient has an underlying lymphoproliferative or myeloproliferative disorder. In the absence of chronic lymphocytic leukemia, chronic myeloid leukemia, or myelodysplastic syndrome, patients should undergo PCR testing for B19 parvovirus DNA [1]. Although not pathognomonic, numerous giant pronormoblasts with absence of maturing erythroid precursors in bone marrow are strongly suggestive of parvovirus-induced PRCA [3,4]. It is crucial to note that nuclear acid amplification testing (NAAT) results for parvovirus B19 DNA detection can be falsely negative, as demonstrated in our patient’s case. Although many PCR-based primer and probe sets have been developed for detecting parvovirus B19-specific DNA targets, most of the oligonucleotides are designed to detect genotype 1 DNA, but not genotypes 2 or 3, which may lead to false-negative NAAT results in those with parvovirus infections from non-genotype 1 variants [6,7]. Of note, the PCR assay used for DNA detection in our case has only been validated for the detection of genotype 1 parvovirus B19 and its ability to detect the less common genotypes 2 and 3 is unknown. Serologic testing with parvovirus B19-specific IgM and IgG antibody assays can be used to support the diagnosis of parvovirus infection in immunocompetent individuals; however, immunocompromised patients are usually not able to generate detectable levels of the antibodies, making serology testing not very beneficial among individuals from the latter population [8]. In a study that looked at the prevalence of parvovirus infection in HIV-positive patients, there was no statistically significant difference in seroprevalence of parvovirus B19-specific IgG between the HIV-positive and HIV-negative cohorts (60.3% vs 68.1%) [9]. Anemia is often multifactorial in etiology in patients with HIV-positive status; however, one study reported that about 25% of severe chronic anemia in HIV-positive patients has been attributed to parvovirus B19 infection [10]. 

Management of PRCA involves supportive care with transfusions, treatment of any underlying disorders, and immunosuppressive or immunomodulatory therapy for individuals who have severe anemia. For hematologic malignancies, systemic therapy with chemotherapy is initiated. With PRCA associated with persistent parvovirus infection, IVIG is often used as part of the treatment regimen as it contains antibodies against parvovirus and can reverse PRCA, with doses as low as 400 mg/kg over 2-5 days [11,12]. Immunosuppressive therapy with cyclosporine or glucocorticoid is generally initiated if anemia or severe reticulocytopenia persists for a month or more [11]. Hematopoietic stem cell transplantation can be considered as a last resort in refractory cases. Common etiologies of death in PRCA include progression of the underlying disorder or its complications (i.e., infection or organ failure). 

## Conclusions

This paper discussed the case of a young male who presented with severe symptomatic anemia. Although the anemia was thought to be secondary to GI blood loss initially, in-depth investigation revealed that he had untreated persistent parvovirus infection in the setting of immunocompromised state leading to PRCA. Administering IVIG to promote antibody formation against the parvovirus, in addition to encouraging future compliance with ART and PPI were key elements in managing our patient’s anemia. This report highlights the importance of keeping parvovirus-induced PRCA in the differential diagnoses when evaluating immunocompromised individuals with severe anemia. Additionally, it underscores the fact that a negative PCR testing for parvovirus B19 DNA does not definitely rule out underlying parvovirus infection and that further investigation through IHC staining can be beneficial, especially when bone marrow biopsy is suggestive of PRCA with near or complete absence of erythroid progenitor cells.

## References

[1] Means RT Jr (2016). Pure red cell aplasia. Hematology Am Soc Hematol Educ Program.

[2] Mangla A, Hamad H (2021). Pure Red Cell Aplasia. StatPearls [Internet].

[3] Brown KE, Anderson SM, Young NS (1993). Erythrocyte P antigen: cellular receptor for B19 parvovirus. Science.

[4] Young NS, Brown KE (2004). Parvovirus B19. N Engl J Med.

[5] Choi Y, Jo JC, Jeon HJ, Kim DW, Chang MH, Kim H (2017). Trend and treatment patterns of aplastic anemia in Korea, pure red cell aplasia and myelodysplastic syndrome in Korea: a nation-wide analysis. Int J Hematol.

[6] Heegaard ED, Panum Jensen I, Christensen J (2001). Novel PCR assay for differential detection and screening of erythrovirus B19 and erythrovirus V9. J Med Virol.

[7] Servant A, Laperche S, Lallemand F, Marinho V, De Saint Maur G, Meritet JF, Garbarg-Chenon A (2002). Genetic diversity within human erythroviruses: identification of three genotypes. J Virol.

[8] Pandey S (2020). Human parvovirus B19 infection in an immunocompromised host. Clin Case Rep.

[9] van Elsacker-Neile AM, Kroon FP, van der Ende ME, Salimans MM, Spaan WJ, Kroes AC (1996). Prevalence of parvovirus B19 infection in patients infected with human immunodeficiency virus. Clin Infect Dis.

[10] Nasir IA, Medugu T, Dangana A (2018). Human parvovirus B19-associated hematopathy in HIV disease: need for clinicopathological revisit. J Biomed Res.

[11] Crabol Y, Terrier B, Rozenberg F (2013). Intravenous immunoglobulin therapy for pure red cell aplasia related to human parvovirus b19 infection: a retrospective study of 10 patients and review of the literature. Clin Infect Dis.

[12] Sawada K, Fujishima N, Hirokawa M (2008). Acquired pure red cell aplasia: updated review of treatment. Br J Haematol.

